# Update on Ghrelin

**DOI:** 10.1155/2010/963501

**Published:** 2010-06-27

**Authors:** Sergueï O. Fetissov, Alessandro Laviano, Satya Kalra, Akio Inui

**Affiliations:** ^1^Digestive System and Nutrition Laboratory (ADEN EA4311), Biomedical Research Institute, IFR23, Rouen 76183, France; ^2^Department of Clinical Medicine, Sapienza University, 00189 Rome, Italy; ^3^Department of Neuroscience, McKnight Brain Institute, College of Medicine, University of Florida, Gainesville, FL 32611, USA; ^4^Department of Behavioral Medicine, Kagoshima University Graduate School of Medical and Dental Sciences, Kagoshima 890-8520, Japan

Few peptide hormones have attracted as much attention of the scientific community as ghrelin, the natural secretagogue for growth hormone (GH) identified by M. Kojima et al. in 1999 [1], resulting in more than 4000 PubMed citations over the last ten years. The initial interest can be attributed to the ability of ghrelin to stimulate feeding in mammals, suggesting it as a potential target for the development of antiobesity drugs. Many studies investigating this issue have however revealed the complexity of the ghrelin system including the differential physiological effects of the three peptide products of the ghrelin gene: *ghrelin*, which is peri-translationally modified via acylation with the octanoic acid; *des-acyl ghrelin* that is not acylated or has lost its fatty acid residue; and* obestatin*, another bioactive peptide derived from the preproghrelin precursor. Most importantly, it has been realized that beside stimulation of GH secretion and increased feeding, ghrelin has multiple biological effects that would be important to preserve if aiming to antagonize ghrelin-mediated positive energy balance. Among these functions, ghrelin was found to regulate gastrointestinal motility and associated sensory functions, to modulate the reproductive and stress axes, mood and emotion, glucose metabolism, as well as affecting the cardiovascular system and renal function.

The present special issue “Update on ghrelin” includes nineteen reviews and one original study and starts with the review by J. D. Veldhuis and C. Y. Bowers who “integrate the GH axis into the ghrelin system” and discuss the biological background of the multiplicity of ghrelin's functions with their relevance to drug development [2]. Other articles, as introduced below, provide in more details some specific aspects of the ghrelin system, altogether providing a comprehensive overview of the current knowledge in the field and showing how ghrelin research has helped advance our understanding of both physiology and disease.

Ghrelin is abundantly synthesized by specialized mucosal cells in the stomach accounting for about 80% of the serum ghrelin production in rats and for 65% in humans. Some ghrelin producing cells are also found in lower parts of the gastrointestinal (GI) tract, where ghrelin expression can be increased, for example, after gastrectomy [3]. Interestingly, gastric ghrelin cells are of the closed type, whereas those in the lower GI tract are of both the closed and the open type, suggesting their regulation preferentially by hormonal or luminal factors, respectively. These cellular and also phylogenic and ontogenic aspects of ghrelin production in the GI tract are reviewed by I. Sakata and T. Sakai [4]. Moreover, Fujimiya and colleagues show that the closed type gastric cells contain ghrelin, des acyl-ghrelin, and obestatin, while the open type cells contain only des-acyl ghrelin supporting a role of luminal pH on des-acyl ghrelin secretion [5].

Although the role of ghrelin to stimulate feeding is evident in mammals, it is possible that this function of ghrelin is evolutionary late and redundant, and that ghrelin has evolved as a messenger mediating other vital functions such as activation of the somatotropic axis. In fact, in fish, as reviewed by S. E. Schwandt et al., the orexigenic effect of ghrelin is an inconsistent finding and can be species dependent while the GH stimulating effect is preserved [6]. This phenomenon is even more evident in birds, where ghrelin stimulates GH secretion and the hypothalamo-pituitary adrenal stress axis but inhibits feeding and drinking. The avian ghrelin system is discussed in details in the review by M. P. Richards and J. P. McMurtry [7].

The integrative role of ghrelin in homeostatic regulation is evident from its function in the regulation of reproduction versus feeding. Ghrelin inhibits the activity of the hypothalamo-pituitary-gonadal axis acting both centrally and peripherally, similar to hypothalamic neuropeptide Y (NPY), a down-stream target of ghrelin. In addition, ghrelin also acts directly to inhibit reproductive function during conditions of energy deficit. The effects of ghrelin in modulation of the activity of the gonadal axis in both males and females are reviewed by J. Dupont et al. [8].

Although ghrelin stimulates feeding, it inhibits drinking and activates neurons of the hypothalamic subfornical organ usually associated with dehydration. This suggests that circulating ghrelin may signal to the brain via the circumventricular organs not necessarily associated with the median eminence. However, how peripheral ghrelin enters the brains is still an unresolved issue discussed in details by M. Fry and A. V. Ferguson who propose the relaying roles of the circumventricular organs such as the subfornical organ and area postrema [9]. 

In its role in food intake regulation, ghrelin interacts with gastrointestinal hormones signaling satiety such as cholecystokinin, bombesin, peptide YY, glucagon-like peptide, pancreatic polypeptide, and amylin. As discussed by A. S. Wisser et al., these peptides may inhibit ghrelin secretion or antagonize its action in the appetite regulatory neurons in the brain [10]. Since plasma levels of ghrelin normally fall after a meal, it is possible to use it as a satiety marker to evaluate the satietogenic properties of different macronutrients for the development of various nutritional antiobesity approaches. How different types of nutrients affect ghrelin secretion is reviewed by C. Koliaki et al. [11].

In addition to the appetite-related neuronal pathways activated by ghrelin, ghrelin receptors are present in many brain areas that can affect mood and emotion, and, indeed, it was found that ghrelin may interfere with the regulation of the stress response, mood, and anxiety. Again, in analogy to the orexigenic NPY, which also regulates these behavioral modalities, the situation with ghrelin is not simple, and both anxiolytic and anxiogenic effects have been attributed to ghrelin as discussed by J. C. Chuang and J. M. Zigman [12]. Further elucidation of the brain ghrelin system should help to clarify the role of ghrelin, which will be important for the designing of drugs targeting the ghrelin system.

Next, five reviews provide full coverage of the important role of ghrelin in influencing gastrointestinal contractility and motility in normal and pathological conditions, as well as showing its relevance to both new and old therapeutic approaches. H. S. Sallam and J. D. Z. Chen introduce the subject with a meticulous review of the experimental and clinical data accumulated during the last ten years related to ghrelin's prokinetic effects [13]. T. Ohno et al. discuss the possibility that changes of plasma ghrelin concentration are not relevant to the prokinetic effect of ghrelin because of the decreased plasma levels found after a meal, similar to the situation observed for motilin, a hormone structurally related to ghrelin [14]. Further, the complexity of the ghrelin system in influencing gastric motility is presented in the review by M. Fujimiya et al. who discuss the differential effects of ghrelin, des-acyl ghrelin, and obestatin, providing evidence for the implication of distinct central pathways in these effects involving hypothalamic NPY, corticotropin-releasing hormone, and urocortin [5]. As gastric motility is altered in subjects with functional dyspepsia who also experience abdominal discomfort, nausea, and decreased appetite, exploring a role of ghrelin in these patients may be worthwhile. Indeed, as discussed by T. Akamizu et al., plasma levels of ghrelin have been found to correlate with reduction of symptoms in these patients and excitedly, a preliminary study using synthetic ghrelin showed a therapeutic potential in functional dyspepsia [15]. Thus, ghrelin or its analogs may become new drugs for the treatment of functional dyspepsia. 

Similarly, improvement in physiological ghrelin secretion may underlie the beneficial effects of other therapeutic approaches, for example, traditional medicine. Such an example is given in the review by T. Hattori who discusses the mechanisms behind the beneficial effects of Rikkunshito, a traditional Japanese medicine based on a mixture of eight herbs. In fact, Rikkunshito was shown to alleviate dyspepsia, for example, during cancer chemotherapy, and this improvement was associated with increased ghrelin secretion [16].

Ghrelin is also expressed in the pancreatic islets and has an intricate relation with insulin in regulation of the glucose metabolism. These effects of ghrelin with relation to the pathophysiology of type 2 diabetes are reviewed in great detail by S. Sangiao-Alvarellos and F. Cordido [17]. The authors also discuss the results obtained in ghrelin- and ghrelin receptor-knockout mice, suggesting that absence of ghrelin signaling is more relevant to glucose homeostasis than to food intake control, supporting the phylogenetic data mentioned above.

Interestingly, although ghrelin stimulates feeding, low plasma levels of ghrelin are commonly present in obesity whereas levels usually increase with weight loss. These data do not support the causative role of ghrelin in increased appetite in obesity but may suggest a link via the role of ghrelin in glucose homeostasis. L. Pulkkinen et al. in their review make an emphasis on the relation between ghrelin and insulin resistance and human genetics in the development of obesity and the metabolic syndrome [18]. Further data of the putative role of ghrelin in obesity come from studies of obese subjects who have undergone bariatric surgery and hence have reduced gastric ghrelin production. As reviewed by D. J. Pournaras and C. W. le Roux [19], ghrelin plasma levels are altered after the surgery, and both decreased and increased levels were documented. In addition, similar results have also been reported in rats [3]. As bariatric surgery is accompanied by reduced appetite and body weight, the serum ghrelin data do not support a causative role of ghrelin in these beneficial effects. However, bariatric surgery is consistently associated with an improved insulin resistance and diabetes, taken by some as a reason to rename the surgery “metabolic” and pointing to that the surgery-associated changes of ghrelin serum levels might still have beneficial effects on the glucose metabolism. 

Expression of ghrelin and the ghrelin receptor was found in vascular endothelial cells providing the background for the vascular effects of ghrelin. In fact, ghrelin was shown to decrease blood pressure, although this effect might also involve the modulation of the central sympathetic tone. The cardiovascular effects of ghrelin are discussed by M. Tesauro et al. showing that activation of the ghrelin system might be a new therapeutic approach for chronic heart failure and cardiac cachexia [20]. A. Laviano et al. review the relevance of ghrelin in another form of cachexia associated with chronic renal failure both in regard to its pathophysiology and to its putative therapeutic role [21]. The authors propose the use of exogenous ghrelin which should be able to overcome endogenous ghrelin resistance present in renal cachexia, that might improve the nutritional status in cachectic patients. In fact, synthetic ghrelin or ghrelin analogs might be considered as a new therapy for a variety of pathological conditions characterized by anorexia or cachexia. For instance, a state of ghrelin resistance is present in anorexia nervosa, and a recent pilot study showed that administration of ghrelin is accompanied by improved appetite in these patients [22]. Another possible indication of “ghrelin therapy” can be anorexia associated with gastrectomy as suggested by the experimental study in Yada's laboratory [3].

To conclude this editorial, ten years of ghrelin research indicate that this peptide initially identified as a GH secretagogue is an universal hormone. As guest editors, we thank all the authors who have contributed to this special issue for preparing excellent articles and we wish the readers a good time in exploring the ghrelin world, which we find ourselves most exciting and promising for the new discoveries and potential therapeutic spin offs.

*Sergueï O. Fetissov* *Sergueï O. Fetissov* *Alessandro Laviano* *Alessandro Laviano* *Satya Kalra* *Satya Kalra* *Akio Inui* *Akio Inui*
